# Insights into the phylogenetic and molecular evolutionary histories of *Fad* and *Elovl* gene families in Actiniaria

**DOI:** 10.1002/ece3.4044

**Published:** 2018-05-04

**Authors:** Joachim M. Surm, Tarik M. Toledo, Peter J. Prentis, Ana Pavasovic

**Affiliations:** ^1^ School of Biomedical Sciences Faculty of Health Queensland University of Technology Brisbane Australia; ^2^ Institute of Health and Biomedical Innovation Queensland University of Technology Kelvin Grove Australia; ^3^ School of Earth, Environmental and Biological Sciences Science and Engineering Faculty Queensland University of Technology Brisbane Australia; ^4^ Institute for Future Environments Queensland University of Technology Brisbane Australia

**Keywords:** Cnidaria, desaturase, elongase, episodic diversifying selection, gene duplication, long‐chain polyunsaturated fatty acid

## Abstract

The biosynthesis of long‐chain polyunsaturated fatty acids (LC‐PUFAs, ≥ C_20_) is reliant on the action of desaturase and elongase enzymes, which are encoded by the fatty acyl desaturase (*Fad*) and elongation of very long‐chain fatty acid (*Elovl*) gene families, respectively. In Metazoa, research investigating the distribution and evolution of these gene families has been restricted largely to Bilateria. Here, we provide insights into the phylogenetic and molecular evolutionary histories of the *Fad* and *Elovl* gene families in Cnidaria, the sister phylum to Bilateria. Four model cnidarian genomes and six actiniarian transcriptomes were interrogated. Analysis of the fatty acid composition of a candidate cnidarian species, *Actinia tenebrosa*, was performed to determine the baseline profile of this species. Phylogenetic analysis revealed lineage‐specific gene duplication in actiniarians for both the *Fad* and *Elovl* gene families. Two distinct cnidarian Fad clades clustered with functionally characterized Δ5 and Δ6 proteins from fungal and plant species, respectively. Alternatively, only a single cnidarian Elovl clade clustered with functionally characterized Elovl proteins (Elovl4), while two additional clades were identified, one actiniarian‐specific (Novel ElovlA) and the another cnidarian‐specific (Novel ElovlB). In actiniarians, selection analyses revealed pervasive purifying selection acting on both gene families. However, codons in the *Elovl* gene family show patterns of nucleotide variation consistent with the action of episodic diversifying selection following gene duplication events. Significantly, these codons may encode amino acid residues that are functionally important for Elovl proteins to target and elongate different precursor fatty acids. In *A*. *tenebrosa*, the fatty acid analysis revealed an absence of LC‐PUFAs > C_20_ molecules and implies that the Elovl enzymes are not actively contributing to the elongation of these LC‐PUFAs. Overall, this study has revealed that actiniarians possess *Fad* and *Elovl* genes required for the biosynthesis of some LC‐PUFAs, and that these genes appear to be distinct from bilaterians.

## 1 INTRODUCTION

The long‐chain polyunsaturated fatty acid (LC‐PUFAs; e.g., ≥ C_20_ molecule) biosynthetic pathway converts PUFAs (e.g., C_18_ molecule) to LC‐PUFAs. Omega‐3 and omega‐6 LC‐PUFAs, such as eicosapentaenoic acid (EPA; 20:5*n*‐3) and arachidonic acid (ARA; 20:4*n*‐6), are converted from PUFA precursors α‐linolenic acid (ALA; 18:3*n*‐3) and linoleic acid (LA; 18:2*n*‐6), respectively. This pathway relies on the action of desaturase and elongase enzymes (Sprecher, 2000).

The fatty acyl desaturase (*Fad*) gene family encodes desaturase enzymes which insert double bonds at different positions of PUFAs. The coordination of multiple functionally different desaturase enzymes is often required to desaturate PUFAs and LC‐PUFAs. Desaturase enzymes are required to have a combination of Δ5 and/or Δ6 activity; however, alternative pathways also exist which utilize desaturase enzymes with Δ8 activity (Cook & McMaster, 2002; Monroig, Li, & Tocher, 2011; Sprecher, 2000). Genes that encode elongase enzymes are from the elongation of very long‐chain fatty acid (*Elovl*) gene family. In mammals, seven members of the *Elovl* gene family have been identified, with different genes encoding elongase enzymes that have altered affinity to elongate precursor fatty acids. Specifically, elongase enzymes encoded by *Elovl1*,* 3*,* 6*, and *7* are involved in the elongation of saturated fatty acids (SFAs) and monounsaturated fatty acids (MUFAs), whereas *Elovl2*,* 4*, and *5* encode enzymes involved in the elongation of PUFAs to LC‐PUFAs (Jakobsson, Westerberg, & Jacobsson, 2006; Leonard, Pereira, Sprecher, & Huang, 2004; Tamura et al., 2009). Despite this research, the distribution and evolution of genes that encode enzymes responsible for the desaturation and elongation of PUFAs remain largely unresolved in many metazoan taxa.

Whole genome and single gene duplication events have played a major role in the distribution and copy number of *Fad* and *Elovl* genes (Carmona‐Antoñanzas, Monroig, Dick, Davie, & Tocher, 2011; Castro et al., 2012; Fonseca‐Madrigal et al., 2014; Kabeya et al., 2017; Li et al., 2017; Li et al., 2010; Mohd‐Yusof, Monroig, Mohd‐Adnan, Wan, & Tocher, 2010; Monroig, Guinot, Hontoria, Tocher, & Navarro, 2012; Monroig, Navarro, Dick, Alemany, & Tocher, 2012; Monroig, Tocher, & Navarro, 2013; Monroig, Webb, Ibarra‐Castro, Holt, & Tocher, 2011; Monroig et al., 2010, 2016, 2017; Morais, Monroig, Zheng, Leaver, & Tocher, 2009; Surm, Prentis, & Pavasovic, 2015). Gene duplication events in mammals have resulted in multiple gene copies encoding desaturase (Fads1, 2, and 3) enzymes, whereas in other vertebrates (such as *Danio rerio*), only a single desaturase, with Δ5 and Δ6 activity, is present (Hastings et al., 2001). Similarly, whole genome duplication events have resulted in the diversification of elongase enzymes observed in vertebrates (Elovl2 and 5) not present in other chordates, which contain a single elongase enzyme referred to as Elovl2/5 (Castro, Tocher, & Monroig, 2016; Monroig et al., 2016). Gene duplication events of *Fad* and *Elovl* gene families have also occurred in a lineage‐specific manner across other bilaterian taxa, such as molluscs (Monroig, Navarro, et al., 2012; Surm et al., 2015). Despite these key observations, the distribution and evolution of *Fad* and *Elovl* genes remain unresolved in early diverging metazoan phyla such as Cnidaria. Furthermore, due to limited molecular studies investigating the *Fad* and *Elovl* gene families in Cnidaria, their phylogenetic and molecular evolutionary histories remain unresolved.

Studies investigating the fatty acid profiles of early diverging metazoan taxa have been focused on the fatty acid profile of cnidarians that rely on an interaction with symbionts, such as *Symbiodinium* (Garrett, Schmeitzel, Klein, Hwang, & Schwarz, 2013; Harland, Fixter, Davies, & Anderson, 1991, 1992; Papina, Meziane, & van Woesik, 2003). From this body of work, there is strong evidence to suggest that the symbionts transfer essential LC‐PUFAs to the host. This was evident with the fatty acid profile of sea anemones that were treated to remove symbionts revealing the presence of LC‐PUFAs, ARA and EPA, but lacked LC‐PUFAS > C_20_ such as docosapentaenoic acid (DPA; 22:5*n*‐3) and docosahexaenoic acid (DHA; 22:6*n*‐3) (Garrett et al., 2013; Harland et al., 1991, 1992; Papina et al., 2003). The fatty acid profile of early diverging metazoan species that lack a symbiotic relationship, however, remains unclear, and further research investigating the ability of these organisms to elongate and desaturate PUFAs to LC‐PUFAs is required.

**Figure 1:**
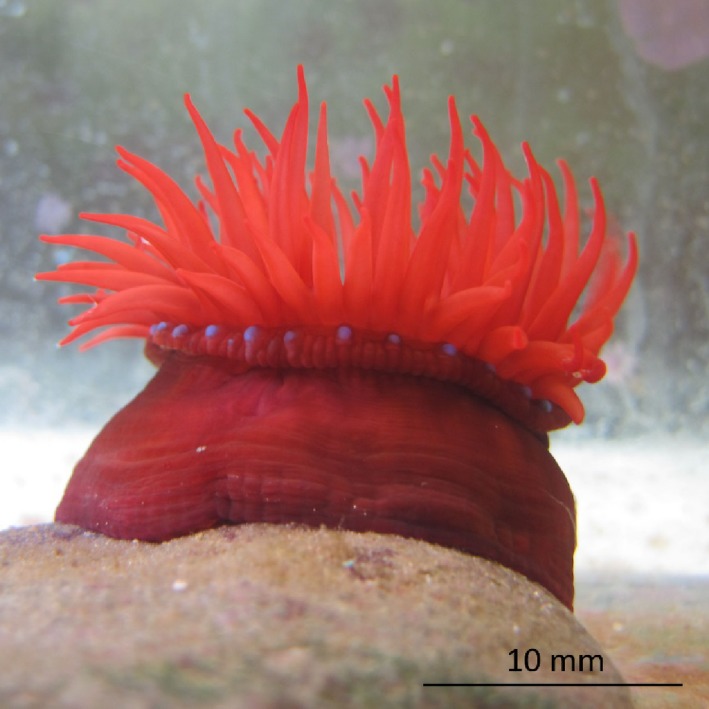
Australian sea anemone, *Actinia tenebrosa*. Photograph credit: Jonathon Muller

Using a comparative genomic approach, this study examined the distribution and copy number of *Fad* and *Elovl* genes from four cnidarian genomes (*Hydra vulgaris*,* Acropora digitifera*,* Nematostella vectensis*, and *Exaiptasia pallida*). A further fine‐scale comparative transcriptomic analysis was also undertaken, within order Actiniaria, to identify specific candidate genes in this group. Phylogenetic and selection analyses of these data have also been performed to elucidate the molecular evolution of the *Fad* and *Elovl* gene families in Cnidarians. The fatty acid profile of candidate cnidarian species, *Actinia tenebrosa* (Figure 1), which lacks a symbiotic relationship with *Symbiodinium* (Black & Johnson, 1979; Muller, Fine, & Ritchie, 2016; Ottaway, 1978), was investigated using fatty acid analysis to address our lack of understanding of the baseline levels of fatty acids in these organisms. Finally, we examined if the fatty acid composition data were concordant with the *Fad* and *Elovl* enzymes found in this species.

## 2 MATERIALS AND METHODS

### 2.1 Identification of candidate genes

*Fad* and *Elovl* candidate genes were identified by interrogating predicted protein sets from a range of species in phylum Cnidaria. The specific species interrogated were *H. vulgaris*,* A. digitifera*,* N. vectensis*, and *E. pallida* (Cnidaria). Furthermore, these genes involved in the synthesis of omega‐3 LC‐PUFAs were investigated in six candidate actiniarian species with sequenced transcriptomes. These species include *A. tenebrosa* (four ecotypes: blue, brown, green, and red), *Anthopleura buddemeieri*,* Aulactinia veratra*,* Calliactis polypus*,* Telmatactis* sp., and *Nemanthus annamensis* from the NCBI Bioproject: PRJNA313244 (van der Burg, Prentis, Surm, & Pavasovic, 2016). All transcriptomic data were generated from either whole organism or multiple tissue types. Raw reads were retrieved from the Sequence Read Archive and converted to FASTQ files. The Trinity software package (v2.0.6) was used to assemble the data after Trimmomatic quality filtering (Bolger, Lohse, & Usadel, 2014; Grabherr et al., 2011). CEGMA was performed to validate the quality, and completeness of the transcriptomes (Parra, Bradnam, Ning, Keane, & Korf, 2009). BUSCO (v3) was also performed using a metazoan‐specific dataset (Simão, Waterhouse, Ioannidis, Kriventseva, & Zdobnov, 2015). TransDecoder version 2.0.1 was used to identify open reading frames (ORF) encoding for proteins to produce a predicted proteome (Haas et al., 2013). CD‐HIT (v4.6.4) was then performed to cluster 100% identical proteins for each individual proteome to remove redundancy (Fu, Niu, Zhu, Wu, & Li, 2012).

Protein sequences generated from both genomic and transcriptomic datasets were then used to identify candidate genes. BLASTp (*e* value < 1e^−05^) was performed using the nonredundant translated ORFs as queries against the Swiss‐Prot database. Potential Fad and Elovl candidates were identified that had a top‐blast hit with a functionally characterized protein from the Swiss‐Prot database (*e* value < 1e^−05^). Functionally characterized Fad and Elovl proteins were identified in the Swiss‐Prot database by having the essential Pfam domains. For Fads, this required Pfam domains: Cyt‐b5 (PF00173) and FA_desaturase (PF00487); and Elovls this required: ELO (PF01151). The respective candidates and functionally characterized Fad and Elovl proteins were aligned using MUSCLE in MEGA 7 (Kumar, Stecher, & Tamura, 2016). Sequences were retained only if they contained essential structural characteristics. These included an N‐terminal cytochrome b5‐like binding domain (cyt‐b5; PF00173), three histidine boxes (HXXXH, HXXXHH, and QXXHH) located in a fatty acid desaturase domain (FA_desaturase; PF00487), and a hem binding motif (HPGG) in Fads. Furthermore, functionally characterized sphingolipid desaturases that also contain these essential structural characteristics were removed from the alignments. The structural characteristics of Elovls included a diagnostic histidine box motif (HXXHH) and a Pfam ELO domain (PF01151). These structural characteristics are essential for desaturation and elongation, and therefore, transcripts not containing these domains were not considered. Candidate genes from actiniarian transcriptomes were checked for symbiont contamination using PSyTranS (https://github.com/sylvainforet/psytrans). The symbiont proteomes from *Symbiodinium microadriaticum*,* Symbiodinium kawagutii*, and *Symbiodinium minutum* were used as a training dataset to identify potential contamination, while the host proteome used for training was *N. vectensis*.

### 2.2 Phylogenetic analyses

The refined list of full‐length translated ORFs was used for phylogenetic analyses to determine the distribution of Fad and Elovl proteins within and across Metazoa. Protein sequences were aligned using MUSCLE in MEGA 7 (Kumar et al., 2016) followed by manual curation to remove sequences that lack conserved residues and motifs. Protein alignments were imported into IQ‐TREE (v1.4.2) (Nguyen, Schmidt, von Haeseler, & Minh, 2014) to determine best‐fit of protein model evolution. Using Bayesian information criterion, a LG+I+G4 model was selected for both Fad and Elovl as the best‐fit model of protein evolution. Phylogenetic trees were generated from alignments using 1,000 ultrafast bootstrap iterations. The Fad tree was visualized using Figtree (v1.4.3) (http://tree.bio.ed.ac.uk/software/figtree/), and the Elovl tree was visualized using Interactive Tree Of Life (v3) (Letunic & Bork, 2016).

### 2.3 Selection analyses

Sequences that encode full‐length protein sequences for both Fad and Elovl proteins generated from actiniarian transcriptomes were investigated to detect the action of pervasive diversifying selection. These codon sequences for the respective *Fad* and *Elovl* gene families were aligned using MUSCLE within MEGA 7 (Kumar et al., 2016). Codon alignments were imported into IQ‐TREE (v1.4.2) (Nguyen et al., 2014) to determine best‐fit substitution model (GTR+I+G4), and phylogenetic trees were generated from alignments using 1,000 ultrafast bootstrap iterations. Using these alignments and phylogenetic trees as inputs, the rates of selection could be determined using maximum‐likelihood models in the program CODEML in PAML (v4.8) (Yang, 2007) using the protocol of (Fang et al., 2009) and codon frequency F3X4. To accurately determine significance, Bonferroni correction was computed to account for the repeated testing of multiple branches (Fletcher & Yang, 2010; Hunt et al., 2011) where *Fad* had *n* = 2 branches and *Elovl* had *n *= 4 branches and the adjusted *p‐*value = .05/*n*. To detect pervasive purifying and diversifying selection, Fast UnconstrainedBayesian AppRoximation (FUBAR) (Murrell et al., 2013) was used from the HyPhy package (Pond, Frost, & Muse, 2005).

### 2.4 Fatty acid analysis

Fatty acid analysis was performed to investigate the baseline fatty acid levels in a candidate cnidarian (*A. tenebrosa*) lacking symbionts. Individuals were placed in isolated holding tank containing only artificial sea water and not fed for 3 days. The fatty acid levels of banana prawn, the primary feed provided to *A*. *tenebrosa*, were also investigated to provide a comparison with the fatty acid profile of *A*. *tenebrosa* prior to starvation. Analysis was performed using three different individuals, with three technical replicates for each individual. An analysis without internal standard was performed to validate the absence of the internal standard (21:0) in the samples.

Lipid extraction was performed using a modification of the method of Matyash, Liebisch, Kurzchalia, Shevchenko, and Schwudke (2008). In brief, 10 mg of the sample was homogenized in liquid nitrogen (LN_2_), immediately an aliquot of 300 μl methanol (cold) containing 0.01% butylated hydroxytoluene, and internal standard (21:0, heneicosanoic acid, 1 mmol/L) (Chem Service INC, West Chester, PA, USA) was added and mixed by vortexing. A 1,000 μl aliquot of methyl tert‐butyl ether was added, and samples were rotated at room temperature for 1 hr. A total of 250 μl of 0.15 mol/L ammonium acetate was added to induce phase separation. Tubes were centrifuged at 2,000 g for 5 min to complete phase separation, and 50 μl of the upper organic layer was removed to a new 2‐ml glass vial and stored at −20°C until analysis, following a similar method utilized by Tran et al. (2014). For the preparation of fatty acid methyl esters (FAMEs), 10 μl of derivatizing reagent (trimethylsulfonium hydroxide solution, ~0.25 mol/L) (Sigma‐Aldrich, Castle Hill, NSW, Australia) was added. The solution was mixed for 30 s and allowed to react for 30 min, following the method proposed by Gómez‐Brandón, Lores, and Domínguez (2010).

The FAME extracts were analyzed using a gas chromatograph coupled to a mass spectrometer (GCMS – TQ8040) (Shimadzu, Kyoto, Japan) with RTX‐2330 capillary columns (Restek, Bellefonte, PA, USA; 60 m × 0.25 mm, film thickness 0.20 μm), and electron ionization set at 70 eV. Conditions for the analysis of FAMEs were as follow: carrier gas, He: 2.6 ml/min; 22:1 split ratio, injection volume 1 μl; injector temperature 220°C; thermal gradient 150–170°C at 10°C/min, then 170–200°C at 2°C/min, then 200–211°C at 1.3°C/min, and temperature held for 5 min. The mass spectrometer was equipped with an ion source (250°C). The data were acquired with Q3 scan mode from *m/z* 50–650. For data collection, the MS spectra were recorded from 4 to 30.5 min.

All data were processed using GCMS Postrun Analysis software (Shimadzu, Kyoto, Japan). FAME identification was based on an internal spectral library as well as a series of FAME standards (20‐component FAME mix) (Restek, Bellefonte, PA, USA) were used to identify retention times (R_*t*_) of specific *m/z* profiles associated with known FAs. The data processing included smoothing, peak detection, integration, peak alignment, normalization, and identification. Extraction and solvent blanks were included in the analysis to allow exclusion of ions detected at lipid masses that result from extraction chemical or solvent impurities. Quantification was achieved by comparison of the peak area of individual lipids to the internal standard.

## 3 RESULTS

### 3.1 Identification of candidate genes

The distribution and copy number of *Fad* and *Elovl* genes found in cnidarian species with sequenced genomes are shown in Table 1. In phylum Cnidaria, gene copy number varies ranging from zero to three *Fad* genes. Genes encoding full‐length Elovl proteins were identified in all taxa, ranging from one to four. From these data, more *Fad* and *Elovl* genes were observed in the actiniarian *E. pallida* compared with the other cnidarian species.

**Table 1:** *Fad* and *Elovl* gene copy numbers in cnidarian taxa with sequenced genomes

Organism	*Fad*	*Elovl*
*Hydra vulgaris*	1	1
*Acropora digitifera*	0	3
*Nematostella vectensis*	1	1
*Exaiptasia pallida*	3	4

The distribution of *Fad* and *Elovl* genes was further investigated in the transcriptome assemblies of six actiniarian taxa, which included *A. tenebrosa*,* A. buddemeieri*,* A. veratra*,* C. polypus*,* Telmatactis* sp., and *N. annamensis*. The six species with sequenced transcriptomes that were used in this analysis are from two superfamilies: Actinioidea (*A. tenebrosa*,* A. buddemeieri*, and *A. veratra*) and Metridioidea (*C. polypus*,* Telmatactis* sp., and *N. annamensis*). The N50 (minimum contig length to cover 50% of the cumulative sum of contigs) for all transcriptomes are > 800 bp and have a completeness > 90% for both CEGMA and BUSCO, with the exception of *Telmatactis* sp. which has a CEGMA and BUSCO completeness of 77% and BUSCO 83.4%, respectively (Tables S1 and S2).

*Fad* and *Elovl* gene copies were identified in all transcriptomes (Table 2). The four *A. tenebrosa* individuals all encode two full‐length Fad proteins, except for the brown individual which encodes a single full‐length Fad protein. Two full‐length Fad proteins are also encoded by *N. annamensis* and *A. buddemeieri*, whereas *C. polypus*,* Telmatactis* sp., and *A. veratra* encode a single full‐length Fad protein. Multiple genes copies encoding full‐length Elovl proteins were also observed in all actiniarian species. All *A. tenebrosa* individuals encode five full‐length Elovl proteins, with the expectation of the brown individual, which encode four full‐length proteins. In the remaining Actinioidea species, *A. buddemeieri* and *A. veratra* encode four and five full‐length proteins, respectively. Metridioidea transcriptomes for *C. polypus*,* Telmatactis* sp., and *N. annamensis* encode five, two, and four full‐length proteins, respectively.

**Table 2:** *Fad* and *Elovl* gene copy numbers in actiniarian transcriptome assemblies

Superfamilies	Organism	*Fad*	*Elovl*
Actinioidea	*Actinia tenebrosa* (blue)	2	5
Actinioidea	*A. tenebrosa* (brown)	1	4
Actinioidea	*A. tenebrosa* (green)	2	5
Actinioidea	*A. tenebrosa* (red)	2	5
Actinioidea	*Anthopleura buddemeieri*	2	4
Actinioidea	*Aulactinia veratra*	1	5
Metridioidea	*Calliactis polypus*	1	5
Metridioidea	*Telmatactis* sp.	1	2
Metridioidea	*Nemanthus annamensis*	2	4

### 3.2 Comparative and phylogenetic analyses of *Fad* and *Elovl* gene families

**Figure 2:**
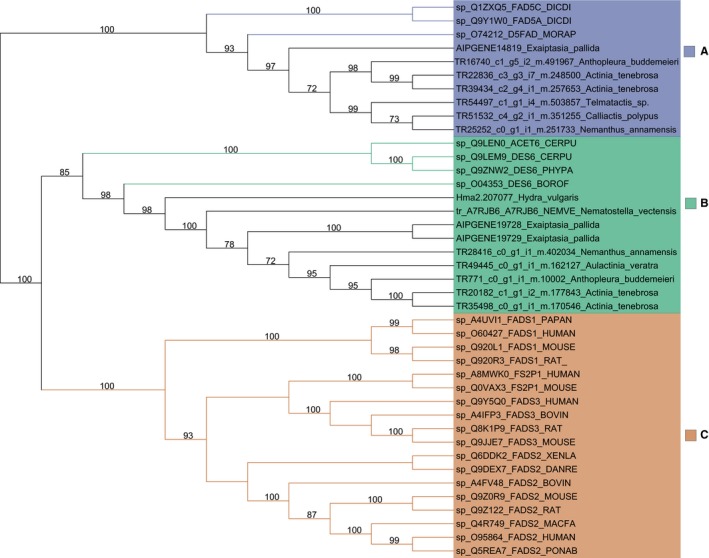
Maximum‐likelihood tree with midpoint root depicting relationships among Fad protein sequences and branches transformed as a cladogram. Bootstrap values after 1,000 iterations are shown next to nodes, values under 70% not reported. Three distinct clades are named clades A, B, and C. The branches of functionally characterized Fad proteins are highlighted by the color of their respective clade. These functionally characterized sequences were retrieved from the SWISS‐prot database and named according to their Uniprot accession with the species name abbreviated, Candidate sequences identified in this study have their full species names

Using a phylogenetic framework, we investigated the distribution of *Fad* genes across Metazoa (Figure 2). A maximum‐likelihood tree revealed three distinct clades, which we name A, B, and C. Clades A and B were sisters to each other, with clade C the most divergent. All bilaterian Fad proteins are found in clade B. Sequences within clades A and C are found to be from phylum Cnidaria as well as non‐metazoan eukaryote taxa, such as fungi and plant species. In fact, functionally characterized plant Fad proteins (green branches) are found in clade A, while functionally characterized fungal and amoebozoan Fad proteins (blue) are present in clade B.

**Figure 3:**
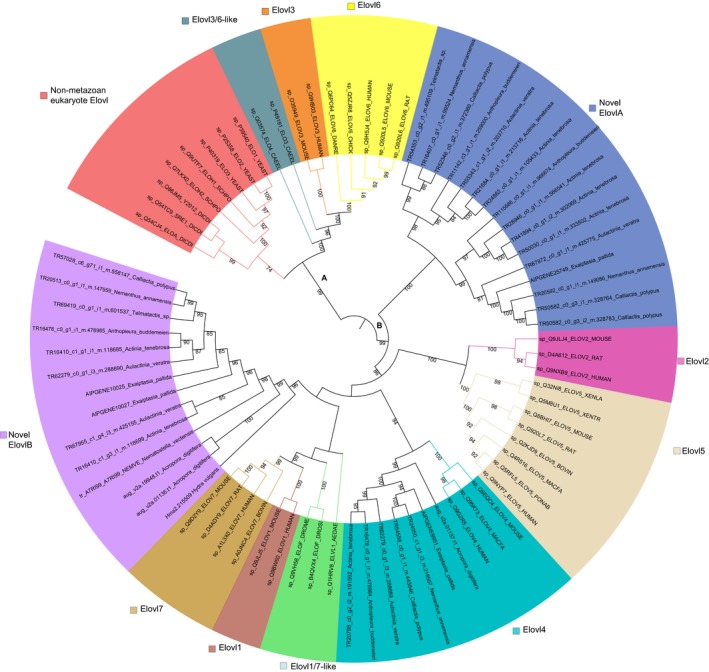
Maximum‐likelihood tree with midpoint root depicting relationships among Elovl protein sequences and branches transformed as a cladogram. Bootstrap values after 1,000 iterations are shown next to nodes, values under 70% not reported. Twelve distinct clades are annotated based on the functionally characterized proteins found within them. The branches of functionally characterized proteins are highlighted by the color of their respective clade. These functionally characterized sequences were retrieved from the Swiss‐prot database and named according to their Uniprot accession with the species name abbreviated. Candidate sequences identified in this study have their full species names

A maximum‐likelihood phylogeny of the *Elovl* gene family produced two clades (A and B) which both contained multiple subclades (Figure 3). Broadly, in clade A, four distinct subclades clustered together including Non‐metazoan eukaryote Elovl, Elovl3, Elovl6, and Elovl 3/6‐like. Sequences from the Non‐metazoan eukaryote Elovl subclade include sequences from *Saccharomyces cerevisiae*,* Schizosaccharomyces pombe*, and *Dictyostelium discoideum*. The Elovl3 clade contains only mammalian taxa and Elovl6 clade contain only taxa from phylum Chordata. The Elovl 3/6‐like clade contains functionally characterized Elovl sequences from *Caenorhabditis elegans* (sp_P49191_ELO3_CAEEL and sp_Q03574_ELO4_CAEEL). A second broad clustering of eight subclades (clade B) can be observed in Figure 3 and was annotated as Elovl1, Elovl7, Elovl1/7‐like, Elovl4, Elovl5, Elovl2, a subclade that include sequences from actiniarian taxa that did not cluster with any functionally characterized sequences (Novel ElovlA), and a subclade that included cnidarian taxa that did not cluster with any functionally characterized sequences (Novel ElovlB). The novel ElovlA subclade was sister to all other subclades in clade B, contained no functionally characterized sequences and consisted only of actiniarian taxa. The Elovl1 and Elovl7 clades were sister to each and contain sequences from phylum Chordata. The Elovl1/7‐like clade contains functionally characterized Elovl proteins from the arthropod *Aedes aegypti* as well as *Drosophila melanogaster* (Chertemps et al., 2007; Ribeiro et al., 2007). The Novel ElovlB subclade consisted of only protein sequences from phylum Cnidaria and no functionally characterized Elovl proteins. This subclade is sister to Elovl1, Elovl7, and Elovl1/7‐like. Subclades Elovl2 and Elovl5 were sister to each other, and only sequences from Chordata are found in these two subclades. In the Elovl4 subclade, both functionally characterized Elovl4 protein sequences, from phylum Chordata, and sequences from phylum Cnidaria clustered together.

### 3.3 Selection analysis of the *Fad* and *Elovl* gene families

To investigate the selection pressures on both the *Fad* and *Elovl* gene families, multiple analyses were performed. Results from the site model selection analysis reveal that both the *Fad* and *Elovl* gene families show patterns of nucleotide variation consistent with the action of pervasive purifying selection (Table 3). The weighted average *d*_N_/*d*_S_ ratio of the *Fad* and *Elovl* gene families for all models is <0.1 for both gene families. Using a chi‐square significance test, the null models, M1, and M7, could not be rejected against the models M2, and, M8, respectively. The null model, M0, however, could be rejected testing against the M3 model, and therefore, the assumption that all codons show the same patterns of nucleotide variation could be rejected. To further examine whether specific codons within gene families are under the influence of pervasive purifying selection or pervasive diversifying selection, FUBAR was used within the HyPHy package (Table S3). These results confirmed no codons are under diversifying selection for both gene families; however, codons were identified to be under pervasive purifying selection. In the *Fad* and *Elovl* gene families, 282 codons of a possible 419 codons, and 197 codons of a possible 223 codons, show patterns of nucleotide variation consistent with the action of purifying selection, respectively. This indicates that pervasive purifying selection is acting on the majority of the codons for both the *Fad* and *Elovl* gene families.

**Table 3:** Detecting pervasive diversifying selection using site models implemented in CODEML for the *Fad* and *Elovl* gene families from actiniarian transcriptome assemblies

Gene families	Model	Likelihood	*d*_N_/*d*_S_	Parameters	Diversifying selected codons
*Fad*	M0 (one ratio)	−8,130.15	0.0814	17	—
	M1 (neutral)	−8,103.01	0.1142	18	—
	M2 (selection)	−8,103.01	0.1142	24	NS
	M3 (discrete)	−8,033.15	0.0935	21	—
	M7 (beta)	−8,041.72	0.0932	18	—
	M8 (beta & ω)	−8,041.61	0.0939	20	NS
*Elovl*	M0 (one ratio)	−9,462.38	0.0795	45	—
	M1 (neutral)	−9,452.13	0.0876	46	—
	M2 (selection)	−9,452.13	0.0876	48	NS
	M3 (discrete)	−9,279.86	0.0856	49	
	M7 (beta)	−9,281.49	0.0845	46	—
	M8 (beta & ω)	−9,281.17	0.0850	48	NS

NS, not significant.

Duplication events have played a major role in the expansion of both gene families in cnidarians, in particular the *Elovl* gene family which has undergone repeated rounds of duplication events. Episodic diversifying selection following duplication events was tested in both the *Fad* and *Elovl* gene families. Maximum‐likelihood trees were constructed using the coding sequence (CDS) for both *Fad* and *Elovl* gene families (Figure S1). From the maximum‐likelihood trees, two subclades in the *Fad* gene family are observed, and four subclades are observed in the *Elovl* gene family. The null hypothesis could not be rejected for any subclade in both *Fad* and *Elovl* gene families, with exception of branch 4 in the *Elovl* gene family which had a *d*_N_/*d*_S_ ratio of 0.003 (Table 4).

**Figure 4:**
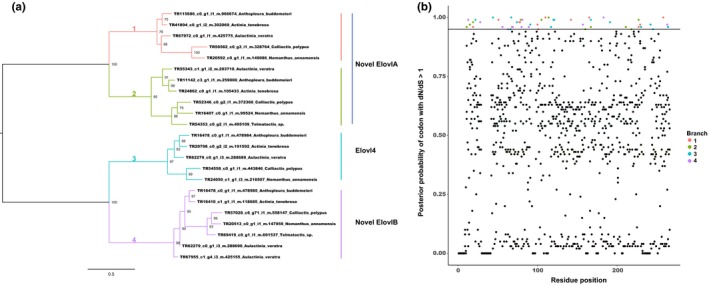
Episodic diversifying selection of *Elovl* gene family in actiniarians. (a) Maximum‐likelihood tree of nucleotide sequences with midpoint root depicting relationships among *Elovl* genes in actiniarians. Foreground branches tested are numbered and colored. Foreground branches corresponding to their respective clades in Figure 3 are annotated accordingly. (b) A plot of posterior probability of codons with *d*_N_/*d*_S_ ≥ 1 against amino acid residue positions. Significantly detected codons under diversifying selection (*d*_N_/*d*_S_ > 1) with posterior probability ≥0.95 (Bayes Empirical Bayes analysis) are colored to correspond to their respective foreground branches. The horizontal line represents the line of significance with posterior probability ≥ 0.95

Finally, the branch‐sites model was implemented to test for codons under episodic diversifying selection following gene duplication (Figure 4). The same foreground branches as previously described were tested (Figure S1). Foreground branches with significant *p*‐values were corrected using Bonferroni correction. The foreground branches that had significant *p*‐values were then used to identify codons with *d*_N_/*d*_S_ ratio > 1 and a posterior probability ≥ 0.95 using Bayes Empirical Bayes (BEB) analysis. The null hypothesis could not be rejected for any of the foreground branches in the *Fad* gene family, and therefore, no codons appear to be under episodic diversifying selection. Conversely, the null hypothesis could be rejected for all foreground branches tested in the *Elovl* gene family. Furthermore, BEB analysis identified multiple codons to be under episodic diversifying selection following duplication events in the *Elovl* gene family. The codons under diversifying selection can be observed in Figure 4 and in Tables S4 and S5. Branch 4, which includes sequences that clustered with functionally characterized Elovl4 proteins (Ohno et al., 2010), was observed to have 15 codons under episodic diversifying selection with a posterior probability ≥ 0.95. The remaining branches are observed to have between 11 and 14 codons under episodic diversifying selection with a posterior probability ≥ 0.95.

### 3.4 Fatty acid analysis

**Figure 5:**
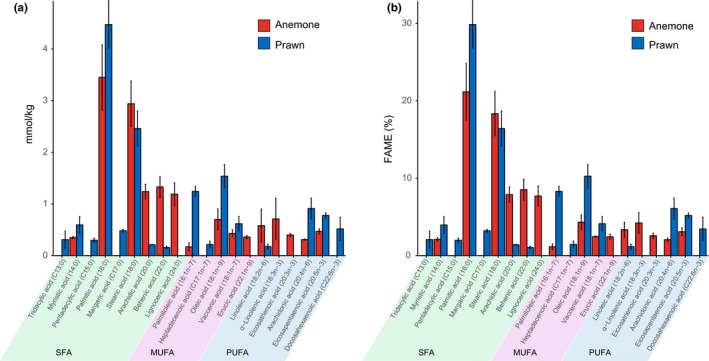
Plot of average fatty acid profile from wholeorganism (*n* = 3) of anemone and prawn. (a) Bar plot of average concentration of fatty acid methyl ester (FAME) given in mmol/kg for both anemone and prawn with error bars shows standard deviation. (b) Bar plot of average concentration of FAME given in % of total FAME for both anemone and prawn with error bars shows standard deviation

Fatty acid analysis was performed for three biological replicates in triplicate to determine the fatty acid profile of an anemone (*A. tenebrosa*) and their feed, banana prawn. FAMEs observed in the whole organism of *A. tenebrosa* and prawn are shown in Figure 5 (Table S6). Of the FAMEs analyzed in *A. tenebrosa*, SFAs are dominant in the samples (65.75% of total FAMEs), followed by PUFAs (15.29% of total FAMEs) and MUFAs (10.37% of total FAMEs). The FAMEs analyzed in prawn also revealed that SFAs are dominant in the samples (59.98% of total FAMEs) followed by MUFAs (24.13% of total FAMEs) and PUFAs (15.89% of total FAMEs).

In *A. tenebrosa* and prawn, SFAs, namely 16:0 and 18:0, are the most abundant component of the total FAME profile (*A. tenebrosa*: 21.17% and 18.34%, respectively; prawn: 29.87% and 16.42%, respectively). Four MUFAs are found in *A. tenebrosa*, and five are found in prawn. In both *A. tenebrosa* and prawn, the methyl ester 18:1*n*‐9 is found in high concentration, 4.32% and 10.27% of total FAMEs, respectively. Multiple different PUFA methyl esters are identified in both *A. tenebrosa* and prawn. In *A. tenebrosa*, LA (18:2*n*‐6), ALA (18:3*n*‐3), ARA (20:4*n*‐6), and EPA (20:5*n*‐3) are present; however, DHA (22:6*n*‐3) was absent. The PUFA methyl esters present in prawn includes LA, ARA, EPA, and DHA. Among the PUFAs, ALA is the methyl ester form most abundant in *A. tenebrosa*, corresponding to 4.22% of total FAMEs, and ARA is the methyl ester form most abundant in prawn, corresponding to 6.09% of total FAMEs (Figure 5).

## 4 DISCUSSION

Research investigating the *Fad* and *Elovl* gene families that desaturate and elongate LC‐PUFAs has largely been restricted to bilaterian taxa (Carmona‐Antoñanzas et al., 2011; Castro et al., 2012; Fonseca‐Madrigal et al., 2014; Kabeya et al., 2017; Li et al., 2017; Li et al., 2010; Mohd‐Yusof et al., 2010; Monroig, Guinot, et al., 2012; Monroig, Navarro, et al., 2012; Monroig, Webb, et al., 2011; Monroig et al., 2010, 2016, 2017, 2013; Morais et al., 2009; Surm et al., 2015). We have investigated the phylogenetic and molecular evolutionary histories of the *Fad* and *Elovl* gene families in phylum Cnidaria, to provide insights into evolution and the distribution of these gene families in Metazoa outside of bilaterian taxa. Our results found multiple copies of both the *Fad* and *Elovl* gene families in cnidarian species, and most of these gene copies had no true ortholog in bilaterian taxa. An expansion of both *Fad* and *Elovl* genes can be observed in actiniarians compared with other cnidarian species. This expansion is the result of lineage‐specific gene duplications in both the *Fad* and *Elovl* gene families. This was evident in both the transcriptomic and genomic data, with the exception of *N. vectensis*. Variations of *Fad* and *Elovl* gene copy number were also observed within the same species as observed in the brown ecotype of *A. tenebrosa*, which had one less *Fad* and *Elovl* compared with the other three ecotypes. This may be an actual case of copy number variation or also could be an artifact of low expression of this gene in this ecotype (Surm et al., 2015).

Actiniarian Fad proteins clustered with functionally characterized Δ5 and Δ6 desaturases Fad proteins in clades A and C, respectively (Figure 2). In clade A, a functionally characterized Δ6 Fad protein with the ability to desaturate PUFAs from the plant species, *Borago officinalis*, is present (Sayanova et al., 1997). In clade C, a functionally characterized Δ5 Fad from the oleaginous fungus, *Mortierella alpina*, was found (Michaelson, Lazarus, Griffiths, Napier, & Stobart, 1998). The fatty acid profile of *M. alpina* has been found to have high levels of ARA and also EPA when conditions are optimal, but lack > C_20_ PUFAs, such as DHA (Knutzon et al., 1998; Michaelson et al., 1998). Furthermore, *M. alpina* has been shown to encode an additional Δ6 desaturase enzyme; however, this sequence is not present in the Swiss‐prot database and therefore was not included in this analysis (Knutzon et al., 1998; Michaelson et al., 1998).

Several sphingolipid desaturases share the same structural characteristics as Fads. These structural characteristics include an N‐terminal cytochrome b5‐like binding domain (cyt‐b5; PF00173), which contains a hem binding motif (HPGG), and a fatty acid desaturase domain (FA_desaturase; PF00487), which contains three histidine boxes (HXXXH, HXXXHH, and QXXHH). To date, these sphingolipid desaturases have been identified in plants, such as *Arabidopsis thaliana* and *B. officinalis*, and fungi such as *Candida albicans* and *Kluyveromyces lactis* (Libisch, Michaelson, Lewis, Shewry, & Napier, 2000; Oura & Kajiwara, 2008; Sperling, Libisch, Zähringer, Napier, & Heinz, 2001; Sperling, Zähringer, & Heinz, 1998; Takakuwa, Kinoshita, Oda, & Ohnishi, 2002). Previous phylogenetic studies (Feng et al., 2017; Gostinčar, Turk, & Gunde‐Cimerman, 2010; Meesapyodsuk & Qiu, 2012) have shown that the paralogs of the genes encoding Fads and sphingolipid desaturases cluster together, as opposed to their respective orthologs. These sphingolipid desaturases that share the same structural characteristics as Fads have yet to be identified in metazoan taxa. Indeed, if these sequences are sphingolipid desaturases, it will be the first report of metazoan sphingolipid desaturase with the same structural characteristics as Fads and would reveal insights into evolution of the Fads and sphingolipid desaturases.

The fatty acid analysis in *A. tenebrosa* revealed a similar fatty acid profile as *M. alpina*, with the presence of EPA and ARA, and an absence of DHA. Although *A. tenebrosa* was starved prior to fatty acid analysis, it is likely that some fatty acids from the diet are incorporated into its lipid profile. This is evident with both *A*. *tenebrosa* and prawn sharing similar lipid profiles; however, this inflated concentration of FAME does not account for the lack of DHA found in the fatty acid profile of *A. tenebrosa*.

In mammals, the *Elovl* gene family has been comprehensively investigated, revealing repeated rounds of gene duplication, resulting in seven members: *Elovl1‐7* (Jakobsson et al., 2006; Leonard et al., 2004). Of these seven genes, only the proteins encoded by *Elovl2*,* 4*, and *5* play a role in the elongation of PUFAs to LC‐PUFAs, with *Elovl1*,* 3*,* 6*, and *7* having roles in elongating other types of fatty acids, such as SFAs and MUFAs. Overall, few orthologs PUFA elongases (*Elovl2*,* 4*,* and 5*) were identified in cnidarians relative to bilaterian taxa. Only bilaterian Elovl4 proteins were found in a clade with actiniarian Elovl proteins. This suggests cnidarians, including actiniarians, lack the diversity of elongases required to biosynthesis LC‐PUFAs. Although chordates are considered inefficient at biosynthesising LC‐PUFAs, their fatty acid profiles contain LC‐PUFAs > C_20_ (e.g., DHA) (Sprague, Dick, & Tocher, 2016). The presence of LC‐PUFAs > C_20_ in bilaterian taxa and their absence in *A. tenebrosa* may be explained by a diversification of the *Elovl* gene family in some bilaterians, resulting in *Elovl2*,* 4*, and *5*. This diversification of PUFA elongases is not present in cnidarians, with only *Elovl4* identified in most species investigated. It should be noted that the Elovl4 protein has been shown to elongate LC‐PUFAs > C_20_ (Castro et al., 2016; Monroig et al., 2013, 2016), and therefore, functional characterization of the Elovl4 protein in cnidarians is required.

In *A. tenebrosa*, our fatty acid analysis results revealed a high proportion of SFAs compared with MUFAs and PUFAs. The higher levels of SFAs, especially the presence of 20:0, 22:0, and 24:0, suggest a capacity of *A. tenebrosa* to elongate SFA from 16:0 and 18:0. In bilaterians, the elongases capable of these SFA elongation are Elovl1, 3, 6, and 7. Furthermore, non‐metazoan eukaryote taxa and early diverging metazoans have somewhat similar fatty acid profiles, with the presence of C_20_ LC‐PUFAs (ARA and EPA) and an absence of LC‐PUFAs > C_20_. The elongase capabilities of the sequences found within both novel subclades (Novel ElovlA and Novel ElovlB) are unknown, as no functionally characterized sequences clustered with these subclades. Currently, the Novel ElovlA and Novel ElovlB subclades appear to be actiniarian‐specific and cnidarian‐specific, respectively. However, including more taxa from other phyla, such as Porifera, Ctenophora, and Placozoa, would be essential in discerning whether the genes from these subclades are lineage‐specific. A lack of DPA and DHA in the fatty acid profile of *A*. *tenebrosa*, however, suggests that the putative function of the proteins encoded by genes that cluster within the Novel ElovlA and Novel ElovlB subclades is unlikely to have an action consistent with those elongase enzymes capable of elongating LC‐PUFAs > C_20_.

Selection analyses of actiniarian *Fad* and *Elovl* gene families revealed significant evolutionary constraint in their CDS, despite repeated rounds of duplications. The *d*_*N*_ */d*_*S*_ ratio for *Fad* and *Elovl* gene families was <0.1 for both, indicating patterns of nucleotide variation consistent with the action of purifying selection. The application of a combination of CODEML and FUBAR identified no codons to be under pervasive diversifying selection; however, FUBAR revealed that the vast majority of codons are under pervasive purifying selection (Table S3). A total of 67% (282/419) and 88% (197/223) of all codons are under pervasive purifying selection for the *Fad* and *Elovl* gene families, respectively. While both gene families were found to be under pervasive purifying selection, some codons were observed to be under episodically diversifying selection in specific clades.

**Table 4:** Detecting lineages under episodic diversifying selection with branch models implemented in CODEML for *Fad* and *Elovl* gene families from actiniarian transcriptome assemblies

Gene families	Branch	H0 Likelihood	H1 Likelihood	*p‐*Value	*d*_N_/*d*_S_
*Fad*	1	−8,130.15	−8,128.74	9.31 e‐02^NS^	NS
	2	−8,130.15	−8,129.28	1.89 e‐01^NS^	NS
*Elovl*	1	−9,462.38	−9,462.37	9.26 e‐01^NS^	NS
	2	−9,462.38	−9,461.54	1.97 e‐01^NS^	NS
	3	−9,462.38	−9,462.01	3.95 e‐01^NS^	NS
	4	−9,462.38	−9,458.16	3.66 e‐03*	0.003

Significance ≤ 0.05 following Bonferroni's correction are highlighted as *. NS, not significant.

The branch‐site model implemented in CODEML identified no evidence of episodic diversifying selection for the *Fad* gene family, whereas strong evidence of episodic diversifying selection was observed following each duplication event for the *Elovl* gene family. Codons in the *Elovl* gene family which were identified to be under episodic diversifying selection may also be responsible for targeting different fatty acids, such as SFA, MUFA, and PUFA. Codons were identified to be under episodic diversifying selection on all four branches tested (Figure 4). From Figure 3, the *Elovl* genes that clustered to the Novel ElovlA and Novel ElovlB subclades appear to be actiniarian‐specific and cnidarian‐specific, respectively. In particular, 15 codons were identified to be under episodic diversifying selection on branch 4 of Figure 4, which corresponds to actiniarian *Elovl* genes orthologs to bilaterian *Elovl4* (Figure 3). As Elovl4 proteins are responsible for elongating PUFAs, these codons may have a role in the targeting and elongating of PUFAs. A study from the genus *Drosophila* revealed that the *Fad* gene family in this group is under the influence of pervasive purifying selection but also episodic diversifying selection at specific codons (Fang et al., 2009). This study also found that the majority of the codons under episodic diversifying selection occurred in clades produced by duplication events. The authors suggest that the amino acid residues under positive selection may be responsible for altered substrate selectivity.

To date, research investigating the ability of metazoan taxa to biosynthesis LC‐PUFAs has been restricted to bilaterian taxa. Here, we provide the first comprehensive analysis of *Fad* and *Elovl* gene families across phylum Cnidaria. Our analysis revealed that lineage‐specific gene duplication has played a major role in the distribution and diversification of both the *Fad* and *Elovl* gene families in actiniarians. The molecular evolutionary histories were investigated revealing pervasive purifying selection for both gene families in actiniarians. However, in the *Elovl* gene family, codons were identified to be under episodic diversifying selection following gene duplication. The amino acids that are encoded by these codons under episodic diversifying selection may be functionally important for targeting and elongating different fatty acids, such as SFA, MUFA, and PUFA. The fatty acid composition data implies that *Elovl* enzymes found in *A. tenebrosa* are not actively contributing to FAs of longer than 20 carbons, but this speculation must be viewed with caution as further functional validation is required before this result is validated Overall, this study has revealed that actiniarian species possess *Fad* and *Elovl* genes required for the biosynthesis of some LC‐PUFAs, and these genes appear to share a greater similarity to non‐metazoan eukaryotes.

## CONFLICT OF INTEREST

None declared.

## AUTHOR CONTRIBUTIONS

AP, JS, TT, and PP conceived and designed the project. JS performed phylogenetic and selection analysis TT performed fatty acid analysis. AP, JS, TT, and PP wrote and edited the manuscript. All authors read and approved the final manuscript.
