# Supplementary figures and images for: Insights into the phylogenetic and molecular evolutionary histories of Fad and Elovl gene families in Actiniaria
Source: Ecol Evol. 2018 May 4;8(11):5323–35. doi: 10.1002/ece3.4044 (PMC6010785; doi:10.1002/ece3.4044)

A

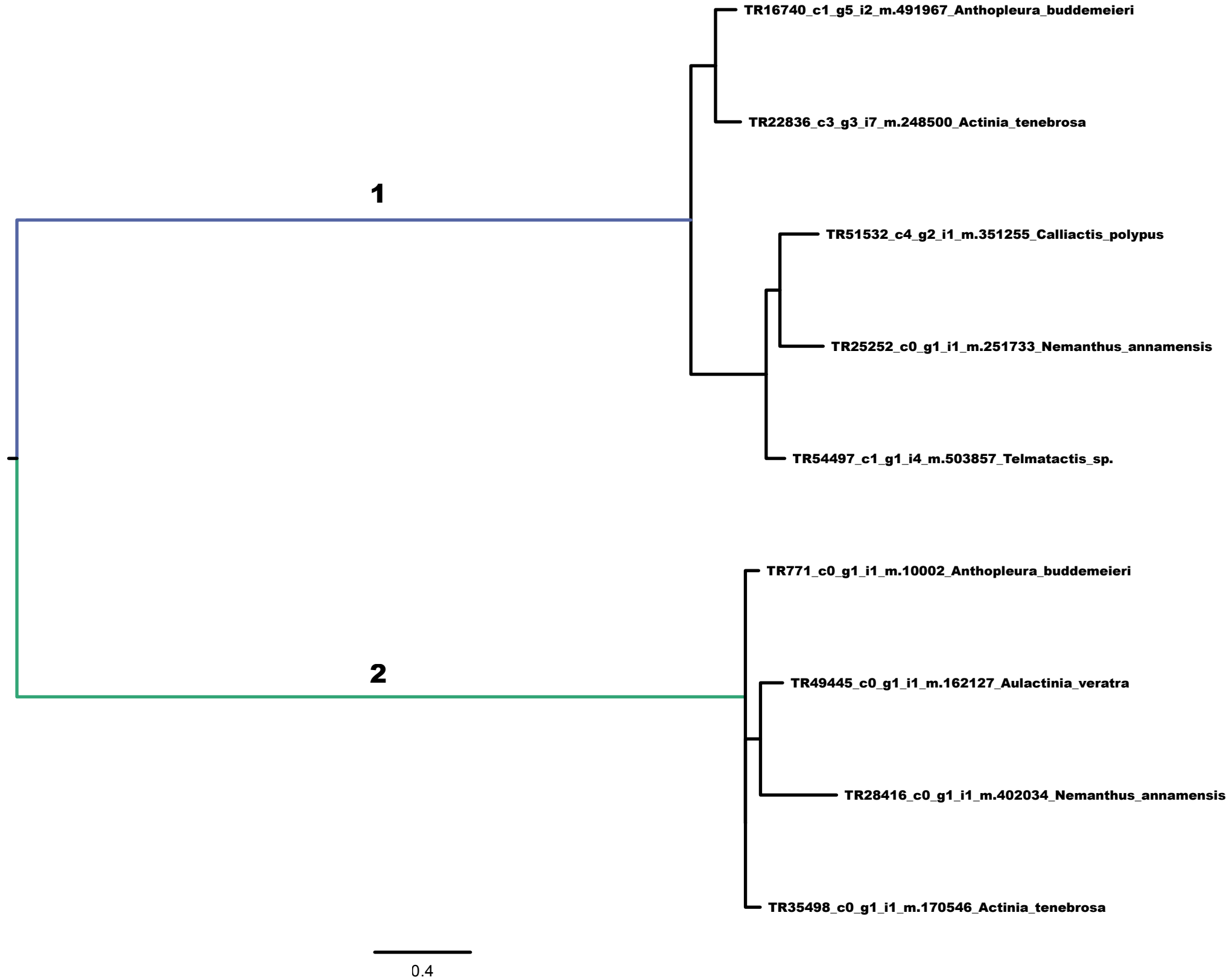

B

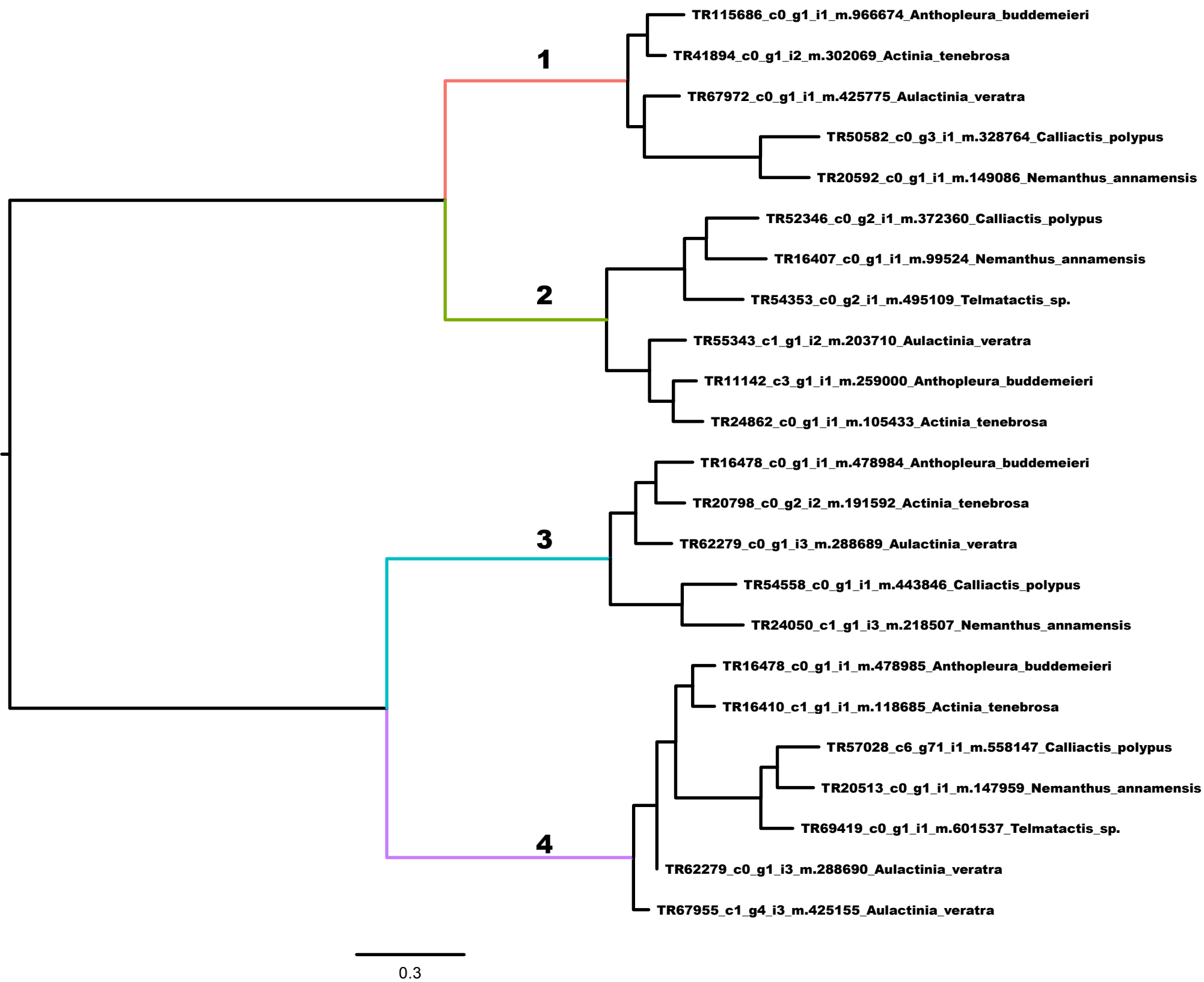

Supplement: Supplementary file 1 [file ECE3-8-5323-s001.pdf]
