# Supplementary material for: Insights into the phylogenetic and molecular evolutionary histories of Fad and Elovl gene families in Actiniaria
Source: Ecol Evol. 2018 May 4;8(11):5323–35. doi: 10.1002/ece3.4044 (PMC6010785; doi:10.1002/ece3.4044)
Supplement: Supplementary file 2 [file ECE3-8-5323-s002.docx]

**Appendix:**

**Table S1.** Transcriptome assembly statistics. Assembly statistics from the Trinity *de novo* assembly of *A. tenebrosa* ecotype (red, brown, blue and green)

| Assembly Metrics | *A. tenebrosa* (ecotype) | | | |
| --- | --- | --- | --- | --- |
|  | Red | Brown | Blue | Green |
| Number of reads | 152,136,760 | 201,995,450 | 175,687,690 | 179,309,262 |
| Total assembled base pairs | 170,903,166 | 179,276,582 | 113,760,033 | 149,624,586 |
| Number of transcripts | 241,041 | 243,560 | 116,930 | 199,049 |
| N10 | 3,875 | 4,609 | 5,192 | 4,517 |
| N30 | 1,992 | 2,345 | 2,889 | 2,325 |
| N50 | 1,084 | 1,237 | 1,804 | 1,264 |
| Average contig length | 709.02 | 736.07 | 972.89 | 751.70 |
| CEGMA (Full length %) | 92.74 | 96.37 | 97.98 | 97.58 |
| BUSCO (% Complete BUSCOs) | 96.3 | 96.9 | 97.3 | 96.9 |

**Table S2.** Transcriptome assembly statistics. Assembly statistics from the Trinity *de novo* assembly of *A. buddemeieri, A. veratra, C. polypus, N. annamensis* and *Telmatactis* sp.

| Assembly Metrics | Species | | | | |
| --- | --- | --- | --- | --- | --- |
|  | *A. buddemeieri* | *A. veratra* | *C. polypus* | *N. annamensis* | *Telmatactis* sp. |
| Number of reads | 51,620,970 | 69,513,111 | 209,875,116 | 205,911,634 | 79,893,721 |
| Total assembled base pairs | 115,667,107 | 175,217,930 | 183,142,054 | 110,971,901 | 114,935,169 |
| Number of transcripts | 332,206 | 264,252 | 225,965 | 116,120 | 190,166 |
| N10 | 2,997 | 3,836 | 5,025 | 5,354 | 3,220 |
| N30 | 1,552 | 1,928 | 2,708 | 2,933 | 1,551 |
| N50 | 851 | 993 | 1,520 | 1,824 | 806 |
| Average contig length | 622.43 | 663.07 | 810.49 | 955.67 | 604.39 |
| CEGMA (Full length %) | 91.13 | 96.37 | 97.98 | 97.58 | 77.02 |
| BUSCO (% Complete BUSCOs) | 95 | 96.9 | 96.7 | 97.3 | 83.4 |

**Figure S1. Maximum Likelihood tree of nucleotide sequences with midpoint root depicting relationships among A) *Fad* genes and B) *Elovl* genes**. Branches are coloured and numbered according to the foreground branches used for testing for episodic diversifying selection.

**Table S3.** Detecting pervasive purifying and diversifying selection using FUBAR(Murrell et al., 2013) within HyPHy(Pond et al., 2005) package at posterior probability of ≥ 0.95

| **Gene family** | | **Number of Codons** | | | |
| --- | --- | --- | --- | --- | --- |
|  |  | *d*_N_/*d*_S_ <1 | | *d*_N_/*d*_S_ >1 | |
| *Fad* | 282 | | 0 | |  |
| *Elovl* | 197 | | 0 | |  |

**Table S4.** Detecting codons under episodic diversifying selection with branch-site models implemented in CODEML for the *Fad* and *Elovl* gene families from actiniarian transcriptome assemblies. Significance at ≤ 0.05 and ≤ 0.01 following Bonferroni's correction are highlighted as * and **, respectively. Codons under episodic diversifying selected detected at ≥ 0.95 significance are indicated and ≥ 0.99 significance in the parenthesis using the Bayes Empirical Bayes analysis. NS refers to not significant.

| **Gene family** | **Branch** | **H0 Likelihood** | **H1 Likelihood** | ***p-*value** | **Diversifying selected codons** |
| --- | --- | --- | --- | --- | --- |
| *Fad* | 1 | -8082.83 | -8081.41 | 9.16 *e*-02^NS^ | NS |
|  | 2 | -8082.83 | -8081.42 | 9.34 *e*-02^NS^ | NS |
| *Elovl* | 1 | -9441.42 | -9435.93 | 9.22 *e*-04** | 14 (5) |
|  | 2 | -9442.85 | -9435.43 | 1.17 *e*-04** | 11 (4) |
|  | 3 | -9443.91 | -9438.58 | 1.10 *e*-03** | 15 (6) |
|  | 4 | -9441.46 | -9434.17 | 1.34 *e*-04** | 11 (3) |

**Table S5.** Codons encoding amino acids under episodic diversifying selection from the branch-site models implemented in CODEML for *Elovl* gene families from actiniarian transcriptome assemblies and a significance at ≥ 0.95 using the Bayes Empirical Bayes analysis. The number refers to the consensus position and letter refers to the amino acid of TR115686_c0_g1_i1_m.966674_Anthopleura_buddemeieri.

| **Gene family** | **Branch** | **Codon under diversifying selection** |
| --- | --- | --- |
| *Elovl* | 1 | 19K, 51I, 68P, 77A, 78L, 100P, 101Q, 110P, 142T, 165L, 211A, 227Q, 229V, 258S |
|  | 2 | 11A, 15M, 19K, 79T, 105D, 114Y, 160A, 167G, 211A, 218T, 227Q |
|  | 3 | 22V, 44A, 66R, 67K, 74F, 91L, 117H, 120Y, 121V, 167G, 191Y, 230G, 242F, 245Y, 264A |
|  | 4 | 13E, 28T, 56W, 63L, 69M, 80S, 81L, 89M, 157M, 183S, 263N |

**Table S6.** Average fatty acid profile from whole-organism (n=3) of anemone and prawn. The concentration of FAME (given in mmol/kg and % of total FAME).

| **Fatty acid** | **Anemone** | | **Prawn** | |
| --- | --- | --- | --- | --- |
|  | **mmol/kg (S. D.)** | **% of FAME (S. D.)** | **mmol/kg (S. D.)** | **% of FAME (S. D.)** |
| Tridecylic acid (C13:0) | 0 (0) | 0 (0) | 0.31 (0.16) | 2.07 (1.08) |
| Myristic acid (14:0) | 0.35 (0.02) | 2.1 (0.23) | 0.59 (0.16) | 3.96 (1.08) |
| Pentadecylic acid (C15:0) | 0 (0) | 0 (0) | 0.3 (0.04) | 1.99 (0.27) |
| Palmitic acid (16:0) | 3.45 (0.63) | 21.17 (3.64) | 4.47 (0.46) | 29.87 (3.05) |
| Margaric acid (C17:0) | 0 (0) | 0 (0) | 0.48 (0.02) | 3.21 (0.16) |
| Stearic acid (18:0) | 2.94 (0.44) | 18.34 (2.86) | 2.46 (0.34) | 16.42 (2.27) |
| Arachidic acid (20:0) | 1.24 (0.14) | 7.89 (0.99) | 0.21 (0.01) | 1.41 (0.05) |
| Behenic acid (22:0) | 1.33 (0.2) | 8.53 (1.36) | 0.16 (0.02) | 1.05 (0.16) |
| Lignoceric acid (24:0) | 1.19 (0.22) | 7.71 (1.28) | 0 (0) | 0 (0) |
| ∑SFA | 10.5 | 65.74 | 8.98 | 59.98 |
| Palmitoleic acid (16:1n-7) | 0.17 (0.08) | 1.15 (0.36) | 1.24 (0.1) | 8.3 (0.64) |
| Heptadecenoic acid (C17:1n-7) | 0 (0) | 0 (0) | 0.22 (0.06) | 1.44 (0.43) |
| Oleic acid (18:1n-9) | 0.7 (0.2) | 4.32 (0.96) | 1.54 (0.22) | 10.27 (1.49) |
| Vaccenic acid (18:1n-7) | 0.43 (0.07) | 2.46 (0.07) | 0.62 (0.14) | 4.12 (0.92) |
| Erucic acid (22:1n-9) | 0.36 (0.03) | 2.44 (0.32) | 0 (0) | 0 (0) |
| ∑MUFA | 1.66 | 10.37 | 3.62 | 24.13 |
| Linoleic acid (18:2n-6) | 0.58 (0.32) | 3.35 (0.95) | 0.17 (0.05) | 1.16 (0.32) |
| α -Linolenic acid (18:3n-3) | 0.71 (0.4) | 4.22 (1.34) | 0 (0) | 0 (0) |
| Eicosatrienoic acid (20:3n-3) | 0.4 (0.03) | 2.57 (0.33) | 0 (0) | 0 (0) |
| Arachidonic acid (20:4n-6) | 0.31 (0.01) | 2.06 (0.21) | 0.91 (0.2) | 6.09 (1.34) |
| Eicosapentaenoic acid (20:5n-3) | 0.47 (0.05) | 3.09 (0.5) | 0.78 (0.05) | 5.19 (0.31) |
| Docosahexaenoic acid (C22:6n-3) | 0 (0) | 0 (0) | 0.52 (0.23) | 3.45 (1.51) |
| ∑PUFA | 2.47 | 15.29 | 2.38 | 15.89 |
| ∑*n*-6 FA | 0.89 | 5.41 | 1.08 | 7.25 |
| ∑*n*-3 FA | 1.58 | 9.88 | 1.3 | 8.64 |
| *n*-6/n-3 | 0.56 | 0.55 | 0.83 | 0.84 |
